# Dysregulation of haematopoietic stem cell regulatory programs in acute myeloid leukaemia

**DOI:** 10.1007/s00109-017-1535-3

**Published:** 2017-04-20

**Authors:** Silvia Basilico, Berthold Göttgens

**Affiliations:** Department of Haematology, Cambridge Institute for Medical Research and Wellcome Trust and MRC Cambridge Stem Cell Institute, University of Cambridge, Hills Road, Cambridge, CB2 0XY UK

**Keywords:** HSPC, MLL gene, AML, ALL

## Abstract

Haematopoietic stem cells (HSC) are situated at the apex of the haematopoietic differentiation hierarchy, ensuring the life-long supply of mature haematopoietic cells and forming a reservoir to replenish the haematopoietic system in case of emergency such as acute blood loss. To maintain a balanced production of all mature lineages and at the same time secure a stem cell reservoir, intricate regulatory programs have evolved to control multi-lineage differentiation and self-renewal in haematopoietic stem and progenitor cells (HSPCs). Leukaemogenic mutations commonly disrupt these regulatory programs causing a block in differentiation with simultaneous enhancement of proliferation. Here, we briefly summarize key aspects of HSPC regulatory programs, and then focus on their disruption by leukaemogenic fusion genes containing the mixed lineage leukaemia (MLL) gene. Using MLL as an example, we explore important questions of wider significance that are still under debate, including the importance of cell of origin, to what extent leukaemia oncogenes impose specific regulatory programs and the relevance of leukaemia stem cells for disease development and prognosis. Finally, we suggest that disruption of stem cell regulatory programs is likely to play an important role in many other pathologies including ageing-associated regenerative failure.

## Regulatory programs in normal haematopoietic stem and progenitor cells (HSPCs)

HSCs reside in the bone marrow, where they represent in the mouse approximately 1 in 20,000 nucleated haematopoietic cells. Though mostly quiescent [1], HSCs actively contribute to steady state haematopoiesis [2], which in turn is largely driven by long-lived multipotent progenitor cells [3, 4]. To maintain a balanced production of the more than ten distinct mature haematopoietic cell types throughout adult life, both HSC self-renewal and multi-lineage differentiation need to be tightly controlled. Despite their rarity, powerful protocols for the prospective isolation of HSCs have been developed, with the latest protocols providing over 60% purity when assayed by single cell transplantation [5]. Nevertheless, their low abundance has been a major obstacle towards the generation of detailed insights into the regulatory programs defining HSC function, because many classical biochemical assays require 1000 or 100,000 of cells that simply are not available for highly purified HSCs. There is renewed hope that new single cell profiling technologies will provide a step-change in our understanding of molecular processes controlling HSC function. This is based on a number of recent publications that (i) defined the transcriptional landscape at single cell resolution [6], (ii) used single cell profiling to train literature-curated network models [7], as well as validating an HSPC regulatory network model built on detailed characterization of gene regulatory sequences [8] and (iii) characterized the transcriptional status of multipotent myeloid precursor cells at single cell resolution [9].

Despite the clear challenges in deciphering regulatory programs operating in HSPCs, some key concepts have nevertheless been identified over the past two decades. Firstly, HSC self-renewal most likely requires extracellular external signals (for example from the bone marrow niche), since HSCs cannot be propagated efficiently in vitro, where a drive to differentiate outstrips any inherent self-renewal capability [10]. Several HSC niches have been proposed with most research focussed on the endosteal [11] and vascular niches [12]. In vivo live imaging [13] partially unified these two concepts by showing that HSCs expansion after BM impairment is taking place in a highly vascularized area where osteoblasts are surrounded by blood vessels. Of note, it has been reported that the majority of HSCs need, for their maintenance, factors secreted by perivascular stromal and endothelial cells [14–16]. Secondly, core circuits of transcription factors define the cellular identity of haematopoietic lineages, such as erythroid, megakaryocyte, or granulocytic cells [17–19]. Multipotent cells, on the other hand, are characterized by low-level co-expression of genes affiliated with distinct lineage programs, such as the co-expression of erythroid and myeloid genes within the same single cells [20–24]. Thirdly, the classical haematopoietic tree with a sequence of binary fate choices is most likely not an accurate reflection of in vivo haematopoietic production, since for example HSCs with restricted or even uni-lineage long-term reconstitution ability have been identified [25–27], and there seem to be multiple differentiation routes that can converge on some of the mature lineages [28]. As with any other biological system, much can be learnt about the properties of HSPCs by studying systemic perturbations. In this context, particular attention has been paid to studying leukaemia models as system-wide perturbations since direct translational relevance will come from a better understanding of the molecular processes that operate in normal HSPCs and are disrupted in leukaemia.

## Perturbation of HSPC regulatory programs in acute myeloid leukaemia

Research during the past 15 years has established that the cell of origin receiving the initial leukaemogenic mutation in acute myeloid leukaemia (AML) is situated within the HSPC compartment [29–31]. Of interest, while full-blown frank leukaemia may be characterized by the expression of surface markers associated with more mature progenitors such as the granulocyte-macrophage progenitor (GMP) [32, 33], it is now thought that the initial mutation more likely occurs within cells residing in the most immature stem and progenitor compartment [34, 35]. Moreover, it has been shown that the ability of an oncogene to transform cells at increasingly mature HSPC stages is oncogene specific [36]. It has been hypothesised that these oncogene specific effects are at least in part related to the degree to which the transforming gene can induce self-renewal in maturing HSPCs that are increasingly distant from the most immature stem cells that naturally possess such activity.

Despite the apparent phenotypic variety of different types of AML driven by both oncogene and cell-of-origin specific effects, common themes have emerged of how HSPC regulatory programs need to be perturbed to cause the leukaemic AML phenotype. This has lead to a classification into types 1 and 2 AML oncogenes: drivers of proliferation or of the block in differentiation, respectively [37, 38]. A combination of types 1 and 2 oncogenes would then create a perfect storm for leukaemia development, as illustrated for example by the combination of activating growth factor mutations (Flt3 or c-Kit) in combination with translocations involving the Runx1 transcription factor. Here, the growth factor mutations cause enhanced proliferation and survival of progenitors, thought to be simultaneously blocked from differentiation by the dominant-negative action of Runx1 fusion proteins [39–42]. However, it needs to be noted that with more detailed molecular characterization of additional AML leukaemogenic mutations, a more complex picture is emerging where individual mutations cannot be classified into strict categories, as they may affect both proliferation and differentiation, and may do so differently depending on which other mutations are present. This suggests that the original model is not only simplistic but in fact may not be useful any longer.

Of particular interest are leukaemogenic mutations commonly associated with childhood acute leukaemias, because it has become apparent that these tumours often have a comparatively low genetic complexity, where two (or even one) genetic hits are thought to be sufficient to cause malignant transformation [43, 44]. Consequently, these diseases offer a more readily interpretable model for the analysis of perturbed HSPC regulatory programs. In the subsequent sections, we will explore in more detail the acute leukaemia cell of origin, and then focus on leukaemogenic perturbations involving the expression of fusion proteins that contain the N-terminal portion of the MLL gene.

## Leukaemia cell of origin

Disease development is characterized by dynamic processes that often begin years before clinical onset. If genetic alterations give survival advantages, the consequence is the growth of a prevalent population, as introduced by Peter Nowell [45] in 1976. Accumulation of sequential somatic mutations in a single clone and subclonal selection lead to the growth of a predominant population with survival advantages. This model has been revisited over the years [46, 47]. For many cancers, the target cell of the transformation events is still unknown. The first evidence for a stem cell origin of cancer was demonstrated by Philip Fialkow in Chronic Myelogenous Leukaemia (CML) [48]. Ten years later, L. J. Smith and colleagues, showed that blast cells from patients with acute leukaemia can co-express markers of both myelopoiesis and lymphopoiesis [32]. The hypothesis for the existence of leukaemia stem cells (LSCs) able to extensively proliferate and sustain leukaemia, came from the observation that proliferation of the majority of leukaemic blasts in AML is finite [49], and only some of the leukaemic cells could form spleen colonies when transplanted in vivo [30]. In the late ‘90s, the use of mouse xenograft models demonstrated that only CD34^+^CD38^−^ human AML purified cells (which can be as low as 0.2% of the total leukaemic cells) were able to repopulate non-obese diabetic mice with severe combined immunodeficiency disease (NOD/SCID mice) and transfer AML. This subset of LSC cells was defined as SCID leukaemia-initiating cells (SL-IC). A significant number of those (2%) retained the CD34^+^CD38^−^ phenotype in vivo confirming their self-renewal property [34]. These observations suggest that for most AML subtypes, immature normal HSPCs rather than committed progenitors, are the target for leukaemic transformation.

However, several studies support an alternative model whereby AML LSCs derive from a downstream progenitor phenotypically identified as CD34^+^CD38^−^CD90^−^ [50]. Of note, fusion transcripts for the AML1-ETO leukaemia oncogene were detected in both blast cells (CD90^−^) and normal HSCs (CD90^+^). However, the latter could differentiate into normal lineage committed cells in vitro without expanding the pool of leukaemic CD90^−^ blasts, indicating that chromosomal translocation may occur in the HSC compartment but transformation into frank AML may require additional mutations that take place in a more downstream population being CD90^−^. Additionally, primary human CD34^+^ AML samples were shown to cluster into two CD90^−^ immunophenotypic groups: GMP-like and more immature LMPP-like cells [51]. Interestingly, gene expression profiles of these LSC populations showed higher similarity with their respective normal counterparts rather than the normal HSC profile.

Further evidence that there is not just a single HSPC regulatory program that is susceptible to leukaemic transformation comes from Acute Promyelocytic Leukaemia (APML) patients where the PML-RARα fusion gene is present in a more differentiated population being CD34^−^CD38^+^ [52]. Jamieson et al., showed that non self-renewing cells were transformed into LSC in human blast crisis chronic myeloid leukaemia (CML), where activation of the Wnt/β-catenin pathway enhanced self-renewal ability of BCR-ABL expressing granulocyte-macrophage progenitors [53]. This study further shows that LSCs could also derive from a differentiated mature cell which can re-acquire self-renewal properties to generate a tumorigenic cell. Furthermore, in vivo transplantation mouse models using primary human MLL-AF4 and -ENL leukaemias have demonstrated that committed progenitors with a CD34^+^CD38^+^CD19^+^ surface marker phenotype were able to give rise to infant ALL [54]. Finally, exciting studies have also provided a definition of the leukaemia cell of origin based on the chromatin landscape, which represents a powerful platform for the identification of new epigenetic biomarkers [55, 56].

However, identification of LSCs cannot be achieved solely by means of cell surface markers, but critically requires functional assays. Thus, understanding the pathways controlling LSCs properties is crucial for the development of new therapies. Oxidative stress variations have been observed to differently correlate with the self-renewal potential of LSC and HSC [57]; inhibitors of the aryl- hydrocarbon receptor (AhR) pathway have been described to block AML cell differentiation [58]; epigenetic inhibitors have been developed to selectively cause apoptosis of AML LSC [59]. Mutations in genes involved in epigenetic processes are indeed involved in the early event of AML evolution, also defined as pre-leukaemic state [60]. These genes are involved in DNA methylation such as DNA methyltransferase 3 alpha (DNMT3A), histones modifications like sex comb like 1 (ASXL1) and chromatin looping like IKAROS family zinc finger 1(IKZF1). Finally, global chromatin changes result also in a complex deregulation of transcriptional programs. It has been recently described that even in the absence of MLL rearrangements, MEIS1 transcriptional program can be activated, as a consequence of its promoter hypomethylation, by DNMT3A mutations [61].

## Cell of origin for pre-leukaemia development

The emergence of subclones after treatment [62, 63] causing relapse has been described in several leukaemias including paediatric leukaemia [64] and CML [65]. Hope and colleagues observed in 2004 proliferation rate heterogeneity in LSCs of AML patients [66]. Quiescence of a subset of LSCs may not only explain the recent discovery that mutations in genes regulating proliferation may be a late event during leukaemogenesis [60], but is also consistent with a model whereby the quiescent normal HSCs are the cell of origin for at least some initiating pre-leukaemic mutations. The earliest clonality studies suggesting the existence of pre-leukaemic HSCs were published by Philip J. Fialkow and colleagues, where X chromosome-linked glucose-6-phosphate dehydrogenase (G6PD) was used as a marker to study clonal remission [67]. At remission, blasts no longer carried the cytogenetic aberration but expressed the “leukaemic” G6PD allele suggesting a leukaemic clonal remission. Long-term remission in AML patients with AML1-ETO translocation similarly showed fusion gene levels in normal single cell derived myeloid and erythroid colonies obtained via isolation of purified HSCs [68]. Further insights into the pre-leukaemic clonal evolution model come from the comparison of DNA copy number abnormalities between diagnostic and relapse samples of paediatric patients with ALL. Fifty-two percent of clones at relapse were antecedent to clones at diagnosis, and the relationship between them was further confirmed via Ig and T cell antigen receptor (TCR) deletions analysis. This unequivocally confirms that the majority of relapse clones derive from a common ancestral one present in small number at diagnosis that acquires additional genetic alterations before emerging as relapse clone [64].

The first study that isolated pre-leukaemic cells in AML patients was published in 2012 [69]. Target exome sequencing was applied on human residual HSC Lin^−^CD34^+^CD38^−^ lacking expression of the AML markers TIM3 and CD99. Even though these cells appeared functionally normal, generating long-term engraftment into NSG mice with both myeloid and lymphoid lineage, they were also demonstrated to be the cellular reservoir causing relapse. In fact, “silent” mutations in critical epigenetic regulators like TET2 were identified in five out of six cases analysed both in purified leukaemic cells and in a fraction of residual HSCs. However, in all cases FLT3-ITD and IDH1 genes were found mutated in AML cells only. Finally, through single cells analysis, sequential acquisition of those mutations in pre-leukaemic cells was demonstrated. Taken together therefore, current evidence suggests that pre-leukaemic cells are closely related to normal HSC and form a cellular reservoir where primary mutations (“silent” mutations) accumulate until secondary events (as FLT3-ITD mutation) confer proliferative advantage that causes frank leukaemia. Perturbation of regulatory programs during leukaemogenesis therefore is likely to be a multi-step process, suggesting that detailed knowledge of the stepwise subversion from normal to pre-leukaemic to leukaemic will be required to obtain a detailed molecular understanding of the underlying processes. As outlined in the previous section, knowing the fusion oncogenes is only part of the story, because the cellular context within which they are activated is equally important.

## MLL-rearranged leukaemia

Leukaemias characterized by chromosomal translocations affecting the MLL gene, encoding a histone H3 lysine 4 (H3K4) methyltransferase, on chromosome segment 11q23 [70], have poor prognosis [71]. MLL rearrangements are responsible for more than 70% of infant (<1 year) leukaemias with either myeloid (AML), or lymphoid (ALL) immonophenotype [43]. MLL translocations occur also in 10% of adult AML [72] and in therapy related acute leukaemias (t-AL), often characterized as tAML, following treatment with topoisomerase II inhibitors [73]. The MLL gene has been found rearranged with multiple partners (more than 50 translocation partners have been identified). Among the most common, MLL-AF9 t(9;11) is mainly associated with AML in both paediatric and adult patients; MLL-AF4 t(4;11) is associated with a lymphoid/mixed-lineage phenotype (mainly ALL), and MLL-ENL t(11;19) drives paediatric ALL and adult AML (only a small fraction of adult patients develop MLL-ENL ALL) [70]. The dismal prognosis of MLL-rearranged (MLL-r) leukaemia is associated with disease relapse [74].

## Retroviral mouse models of MLL-r leukaemia

Several mouse models bearing MLL fusion proteins have been developed in order to understand MLL fusion mediated leukaemogenesis. However, discrepancies between mouse leukaemic models and human leukaemias in terms of ability of MLL fusions to generate the same lineage leukaemia (AML or ALL), as observed in patients, and latency in leukaemia development, make the deconstruction of MLL-r leukaemia development a challenge. One of the first mouse leukaemic models with MLL fusion, was described by Lavau et al. in 1997 [75]. Retroviral transduction of MLL-ENL into lineage-depleted or c-kit sorted mouse bone marrow (BM) HSPCs was followed by culture in methylcellulose. Infected haematopoietic progenitors maintained self-renewal potential in vitro. MLL-ENL expressing progenitors were phenotypically immature myelomonocytic cells being c-kit^+^, Mac1^+^ and Sca-1^−^, and when cultured with granulocyte colony-stimulating factor (G-CSF), terminally differentiated in mature granulocytes. Moreover, their injection into SCID mice, caused death due to AML development. However, while the MLL-ENL translocation gives rise also to human ALL [70], no lymphoid markers were expressed on MLL-ENL expressing cells in the retroviral mouse model. More recent studies demonstrated transforming ability of MLL fusions (both in vitro and in vivo) also in more differentiated haematopoietic progenitor cells [76] such as common myeloid progenitors (CMPs) and granulocytic/monocytic-restricted progenitors (GMPs). By contrast, megacaryocytic/erythroid-restricted progenitors (MEPs) injected mice did not develop AML. Overall, this suggests that MLL-ENL is able to confer a self-renewal regulatory program to some but not all committed progenitors [77]. Subsequent genome wide studies demonstrated that MLL-ENL transcriptional reprogramming happens fast during transformation, and the immediate phase of leukaemogenesis is similar to the progression phase characterized not only by HoxA cluster up-regulation but also by general transcriptional downregulation involving key haematopoietic transcription factors Gata2, Gfi1b and Zfpm1 [78].

Of note*,* murine models [76, 79, 80] often fail to reproduce the biphenotypic feature observed in MLL-r leukaemia patients with co-expression of some myeloid and lymphoid genes. Zeisig et al. [81] first reported a MLL-ENL transformation model based on a biphenotypic lymphoid/myeloid phenotype. Similarly, when infected murine BM cells were cultured in methylcellulose with Flt3-ligand, stem cell factor (SCF) and interleukin-7 (IL-7) to sustain lymphopoiesis [82], B220^+^CD19^+^ and B220^+^CD19^−^ B cells appeared after 4 weeks. The latter generated leukaemia in vivo characterized by splenomegaly, lymph node enlargement and an overgrown thymus, where cells showed a myeloid morphology, yet expressed the B220 lymphoid marker.

## Non-retroviral models of MLL-r leukaemia

All the studies described so far used retroviral models, which may not generate expression levels representative of the endogenous gene loci involved in the translocation events. Since expression levels are critical determinants of cellular programming, it is not surprising that constitutive and conditional knock-in mouse models have provided another powerful approach to analyse MLL-r leukaemias. In 1996, Corral et al. [83] engineered expression of the MLL-AF9 oncogene via homologous recombination [84]. Engineered mice, bearing the MLL-AF9 fusion, developed leukaemia restricted to the myeloid lineage despite of the widespread expression of the fusion gene. Moreover, AML development was characterized by long latency suggesting the need of genetic alterations for complete leukaemic transformation. A Cre-Lox recombination approach generated MLL-AF9 [85] and MLL-ENL [86] mouse models able to rapidly develop AML. Furthermore, de novo MLL-ENL translocations caused myeloproliferative-like myeloid leukaemia development in all mice in which Cre recombinase was expressed from Lmo2, Lck and Rag1 genes (expressed in non-differentiated cells, T-cell linage and early staged of lymphoid lineage, respectively); while no haematological malignancies were observed in MLL-ENL Cd19-Cre (gene expressed in B cell lineage) [87]. Overall, these data demonstrate that the MLL-ENL fusion is leukaemic when expressed in stem cells and progenitors excluding the B-cell compartment [87, 88].

Importantly, an endogenous knock-in mouse model using the MLL-AF9 oncogene [89], demonstrated that GMPs were refractory to leukaemic transformation in complete contrast to previous retroviral studies [76, 79]. While only 100 HSCs and 2500 CMPs from knock-in mice were able to produce AML in the majority of the recipients, all mice transplanted with higher doses of GMPs did not develop any disease. The ability of GMPs to be transformed in retroviral studies seems to be related to the different levels of oncogene expressed (170-fold higher then knock-in GMPs). By contrast, a doxycycline (Dox) inducible mouse model, targeting MLL-ENL to the 3′ UTR of the Col1a1 gene [90], showed that both HSCs and MPPs failed to induce leukaemia in vivo [33]. AML development was observed only when Dox was administrated to mice 4 weeks after HSC transplantation suggesting that MLL-ENL expression could interfere with in vivo homing. The authors therefore suggested that granulocyte-monocyte-lymphoid progenitors (GMLPs), as a subpopulation of the wider Lineage^−^Sca-1^+^c-kit^+^ (LSK) population [91], and GMP precursors (pGMs) represent the most permissive cellular environment for regulatory program perturbations that can cause leukaemia development. Nevertheless, a recent study published in 2016 [92] using another MLL-AF9 Dox inducible mouse model (67), showed that both long-term HSCs (LT-HSCs) and GMP were transformed by MLL-AF9 induction, where transformation in LT-HSCs resulted in a more aggressive AML phenotype. Taken together, rather than providing conclusive answers to the molecular processes underlying AML development in patients, these studies further highlight the intricacies of perturbing regulatory programs and the complex interplay of parameters such as cellular context and oncogene expression level. Given that no mouse model seems perfect, it may be argued that research efforts need to be refocused onto molecular studies with human patient samples, especially since genome engineering has become so much easier with the new clustered regulatory interspaced short palindromic repeats (CRISR) system [93].

## Concluding remarks

**Fig. 1.**
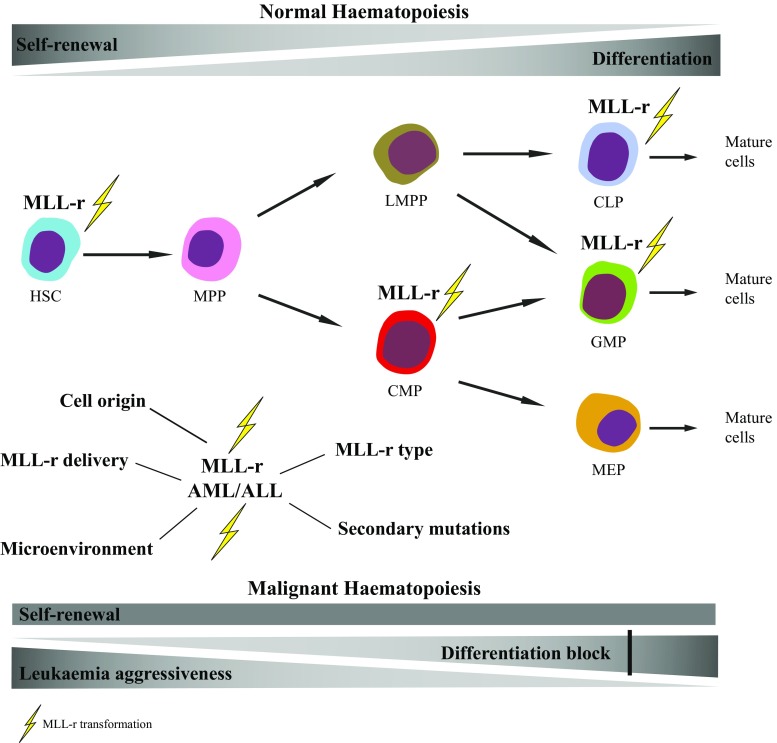
Perturbation of haematopoietic development by MLL rearrangements (MLL-r). MLL-r impair self-renewal and differentiation properties of HSCs and HSPCs. MLL driven leukaemic transformation has been mainly described in HSC, CMP, GMP and CLP [31, 33, 75, 76, 81, 86, 87, 92]. MPP and LMPP progenitors are also targets of MLL transformation as cellular permissiveness might be influenced by the specific strategy used to purify HSPCs [5, 91]. Biology and prognosis of AML and ALL bearing MLL translocations depend on multiple factors: cell of origin of leukaemic transformation, type of MLL-r, oncogene delivery methods, microenvironment and secondary mutations.

Regulatory programs in HSPCs need to be finely balanced to maintain normal haematopoiesis and are vulnerable to genetic perturbations that result in the development of malignant disease. Despite the remaining disagreements between the various retroviral and transgenic models, it is clear that the cell of origin influences leukaemia biology and prognosis (see Fig. 1). Secondly, even within a group of related oncogenes such as all the MLL fusions, the type of MLL translocation together with the nature of the cellular environment strongly influences leukaemia onset and phenotype [54, 88].

Further difficulties in understanding this interplay come from inconsistencies between human disease and the murine models. MLL-ENL expressing cells give rise to AML in mouse models [75, 76, 94] while in human patients, this translocation is mainly involved in paediatric ALL [70]. Moreover, mouse models reconstructing MLL-AF4 ALL have been difficult to develop, which may at least partly be caused by limitations of retroviral technology [95]. While MLLAF4 is associated with paediatric and human ALL [70] and can cause transformation of early B -cells, constitutive knock-in mouse models and mice conditionally expressing MLL-AF4 fusion develop only mature B-cell lymphomas [96, 97].

Overall, these discrepancies are likely to not only be a consequence of intrinsic differences between the human and the mouse system but also because of the many variables associated with both in vitro and in vivo studies. Cellular permissiveness might be influenced by the specific strategy used to purify HSPCs [33] as well as oncogene delivery methods [91, 92]. Moreover, a preferential association of some MLL fusions with specific leukaemia subtypes could be under microenvironmental influence or dependent on cytokine signalling sensitivity [98]. The involvement of secondary mutations associated with MLL translocations will have major impacts on the way regulatory programs are perturbed, and therefore will be linked with the aggressiveness of the disease. Although MLL-r AML had been considered to have a low mutation frequency [44], some MLL leukaemia models show long latency preceded by a pre-leukaemic phase [75, 99]. Accordingly, a recent cancer genome sequencing study on MLL-r AML patients has identified mutations in SPI1gene [100], a powerful HSPC transcription factor whose role in murine AML had already been defined at genomic scale [101].

Much remains to be learnt about the complex regulatory programs that are responsible for stem cell function during both tissue maintenance and repair. The haematopoietic system offers exciting opportunities to not only define these processes in normal cells, but also to learn how system perturbations can lead to disease development. While current research efforts are largely aimed at improving our understanding of perturbations that corrupt normal HSPCs towards creating a malignant state, many of the underlying principles will be widely applicable to other instances of stem cell state subversion. In non-haematopoietic tissues, adult stem cell deficiencies, particularly in old age, are associated with both imbalances in tissue maintenance as well as regenerative failure. A better understanding of the mechanisms that underlie the corruption of stem cell regulatory programs is therefore of broad therapeutic relevance [102].
